# Genome-Wide Association Analysis of Radiation Resistance in *Drosophila melanogaster*

**DOI:** 10.1371/journal.pone.0104858

**Published:** 2014-08-14

**Authors:** Mahesh Vaisnav, Chao Xing, Hung-Chih Ku, Daniel Hwang, Strahinja Stojadinovic, Alexander Pertsemlidis, John M. Abrams

**Affiliations:** 1 Department of Cell Biology, UT Southwestern Medical Center, Dallas, Texas, United States of America; 2 McDermott Center for Human Growth and Development, UT Southwestern Medical Center, Dallas, Texas, United States of America; 3 Department of Radiation Oncology, UT Southwestern Medical Center, Dallas, Texas, United States of America; 4 Greehey Children’s Cancer Research Institute, Departments of Pediatrics and Cellular & Structural Biology, UT Health Science Center at San Antonio, San Antonio, Texas, United States of America; University of Iceland, Iceland

## Abstract

**Background:**

Ionizing radiation is genotoxic to cells. Healthy tissue toxicity in patients and radiation resistance in tumors present common clinical challenges in delivering effective radiation therapies. Radiation response is a complex, polygenic trait with unknown genetic determinants. The *Drosophila* Genetic Reference Panel (DGRP) provides a model to investigate the genetics of natural variation for sensitivity to radiation.

**Methods and Findings:**

Radiation response was quantified in 154 inbred DGRP lines, among which 92 radiosensitive lines and 62 radioresistant lines were classified as controls and cases, respectively. A case-control genome-wide association screen for radioresistance was performed. There are 32 single nucleotide polymorphisms (SNPs) associated with radio resistance at a nominal *p*<10^−5^; all had modest effect sizes and were common variants with the minor allele frequency >5%. All the genes implicated by those SNP hits were novel, many without a known role in radiation resistance and some with unknown function. Variants in known DNA damage and repair genes associated with radiation response were below the significance threshold of *p*<10^−5^ and were not present among the significant hits. No SNP met the genome-wide significance threshold (*p* = 1.49×10^−7^), indicating a necessity for a larger sample size.

**Conclusions:**

Several genes not previously associated with variation in radiation resistance were identified. These genes, especially the ones with human homologs, form the basis for exploring new pathways involved in radiation resistance in novel functional studies. An improved DGRP model with a sample size of at least 265 lines and ideally up to 793 lines is recommended for future studies of complex traits.

## Introduction

Ionizing radiation is genotoxic to cells. The acute effects of whole body irradiation depend on several factors, among which cell type and total dose are most prominent. Whole body exposure to radiation is implicated in gastointestinal syndrome and hematopoietic syndrome, and for doses higher than 30 Gy, cardiovascular collapse, central nervous system damage and death within 24–72 hours [1]. Direct cellular response to radiation involves formation of complex DNA double strand breaks, leading to mutations and genomic instability that may cause cancer [2]. The progeny of irradiated human and murine cells have been demonstrated to develop chromosomal abnormalities or other mutations after multiple generations, and these genomic changes are the same as those observed in human tumors [3]. Thus, there are strong genetic components involved in radiation response. Interestingly, there is no evidence for radiation-induced germline mutations or heritable genetic diseases in children of irradiated parents [4], [5], [6]. In comparison, somatic cell radiosensitivity has been established to be a heritable trait in humans [7], [8].

It has been estimated that approximately 80% of inter-patient variation in normal tissue toxicity following radiation exposure is likely to be genetic, whereas only 20% of the variation results from stochastic events associated with the random nature of radiation-induced cell killing in addition to random variations in dosimetry and dose delivery [9]. Studies addressing this have mainly focused on candidate genes, however they have failed to demonstrate unequivocal links between genotype and radiation toxicity [10].

Radiotoxicity, also known as radiosensitivity, is thought to be a polygenic trait involving interactions of multiple loci involved in different cellular pathways, most of which confer a relatively small risk [10], [11]. To date, three human genome-wide association (GWA) studies have been conducted on radiation response, leading to the discovery of two new genes, *FSHR* and *PRDM1,* involved in radiotoxicity [12]. However, human GWA studies suffer from a number of limitations, the chief one being the inability to identify causal variants [13], [14].

The *Drosophila* Genetic Resource Panel (DGRP) is a powerful community resource for interrogating heritable, natural variation and linking complex traits to underlying genotypes [15]. Sponsored by the National Human Genome Research Institute, this open platform contains 192 fully sequenced inbred *Drosophila* strains harboring nearly 5 million single nucleotide polymorphisms (SNPs). As a discovery platform, the DGRP offers compelling advantages for understanding how genes specify complex traits. First, all sequence data, SNP calls, stocks and associated web-based tools are publicly available to the research community. Second, unlimited sampling of these highly inbred strains can dramatically magnify statistical power. As experimental surrogates for individual variation, DGRP stocks collectively deliver a much higher statistical power compared to outbred individuals [16]. Third, nucleotide level resolution delivers sequence variants that are more likely to be causative [15], which distinguishes it from human GWA studies that delivers SNPs that are usually linked to an unknown culprit. A final advantage afforded by the DGRP draws on the power of the model itself. As a genetic system, *Drosophila* offers opportunities for follow up examination of candidate variants. Taken together, these capabilities facilitate unbiased discovery and functional examination of natural sequence variants that determine traits of interest.

Here, we conducted GWA mapping of radiation resistance in the DGRP lines by assaying survival of adult males at a fixed radiation dose.

## Materials and Methods

### Drosophila stocks

We used 154 inbred lines of the DGRP [15], available from the Bloomington Drosophila Stock Center. Flies were reared at a controlled density on cornmeal-molasses-agar medium at 25°C, 60–75% relative humidity and a 12-h light-dark cycle.

### Experimental setup

The radiation source was a commercial Cs^137^ irradiator (J. L. Shepherd, Model Mark I-68A, Serial Number 1158, San Fernando, CA) emitting 662 keV gamma photons. The irradiator was calibrated using a PTW N31010 ionization chamber (PTW – New York Corporation, Hicksville, NY). The air-kerma calibration coefficient for Cs^137^ photons was obtained from the Accredited Dosimetry Calibration Laboratory (ADCL) at the University of Wisconsin. In addition to the absolute dose rate calibration at a reference point, a relative isodose distribution within the irradiator was obtained using radiochromic films (Gafchromic EBT3, International Speciality Products, Wayne, NJ). The flies were irradiated in cylindrical vials with an inner diameter of 2.3 cm, where the lower part of the vial typically had 2.5 cm of food and the flies were able to fly within 5 cm above that. The vials were placed at the bottom of a rotating plate and the plate was kept rotating the whole time during irradiation. The average dose rate in the vial was 4.85 Gy/min, resulting in a total dose of 1382 Gy over the 4 hours and 45 minutes of continuous exposure.

### Radiation assay

We developed and optimized a radiation toxicity assay using the *Canton S* strain, controlling for the effects of age (7–11 days old), sex (males only), number of flies per trial (n = 50) and survival phenotype endpoint (24 hours post-irradiation). We also found no noticeable difference in vial temperature before and after irradiation, eliminating resistance for heat stress as a potential confounder.

We used the assay in a pilot experiment to test for its ability to induce variation in response in 10 randomly chosen DGRP lines. We then measured the radiation response of the remainder 144 lines, and collected data for two replicates per line. For variable lines, we performed up to 7 replicates per line. Survival was defined as the ability of males to fly 24 hours post-irradiation, and survival time was expressed in percentage as the mean of two trials.

### Phenotypic stability

For temporal phenotypic stability experiments, 12 highly resistant DGRP lines were maintained for five to fourteen months under normal rearing conditions, and were reassayed for radiation resistance using the identical assay conditions.

### Extreme phenotype line crosses

Reciprocal matings were set up between completely sensitive line RAL-28 and extremely resistant line RAL-69, and F1s were selfed to generate F2s. Both F1s and F2s were aged 7–11 days and their radiation response measured under identical assay conditions.

### Genome-wide association analyses

**Figure 1:**
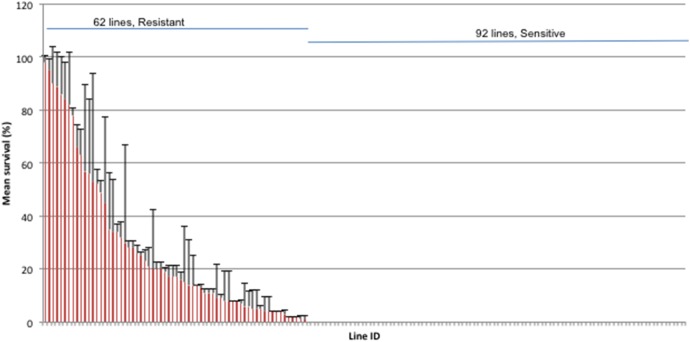
Variation in radiophenotype among 154 DGRP lines. 50 males from each line were aged for 7–11 days, irradiated with 1382 Gy and the number of survivors scored 24 hours post-irradiation. The data shown is the mean of two independent trials; the error bars represent standard deviation. The 62 resistant and 92 sensitive lines are indicated by blue lines.

We treated radiation response phenotype as a binary outcome – resistant and sensitive – since 60% of the data had numerical values of zero (Figure 1). A subset of resistant lines was highly variable (see Results). However, variability did not affect our data analysis because variable lines exhibited a mean resistant phenotype after several replications. Similarly, although the temporal phenotypic stability analysis also showed variability in highly resistant lines, no highly resistant line became sensitive. Hence, variability in the resistance phenotype did not alter the number of cases and controls for association analysis.

There were a total of 5,066,519 SNPs in the DGRP freeze 1 dataset. Genotype data were cleaned by the following criteria: genotype missingness <15%, heterozygous haploids and genotype call rate >10% and minor allele frequency >1%. There were 2,035,449 SNPs filtered out by quality controls.

Potential confounding factors - *Wolbachia* infection status, population stratification, and cryptic relatedness – were considered. *Wolbachia* infection status had no significant effect (Table S3). To examine whether population structure is an influential confounder, we first derived the top five principal components using all the 2,035,449 SNPs by GCTA [17]; then we tested association between radio resistance and each principal component. The results suggested population structure not a significant confounder (Table S1). To thoroughly control for population stratification and cryptic relatedness, we employed a linear mixed model that uses the whole-genome data to estimate the genetic relationship matrix, as well as the top five principal components as covariates [18]. In sum, the association of radiation response in 154 lines with 3,030,570 SNPs was examined by the likelihood ratio test [19] fitting a linear mixed model using GEMMA [20].

To determine the threshold for genome-wide significance, we calculated the number of haplotype blocks in the DGRP genome. The haplotype block is defined as a window of SNPs with the outer-most marker required to be in strong linkage disequilibrium (LD) with an upper limit of 90% confidence interval exceeding 0.98 and a lower limit of 90% confidence interval exceeding 0.7 [21]. The calculation was performed using PLINK [22]. Note that the pairwise LD was only calculated for SNPs within 500 kb; thus the number of LD blocks obtained would be an upper limit. The narrow sense heritability from additive genetic effects was estimated by a liability threshold model using the whole-genome variation [23]. We also estimated heritability by fitting traditional linear mixed models modelling line effects.

## Results

### Inbred strains exhibit natural variation in radiation response

To characterize natural variation in radiation response in 154 DGRP lines, we selected a fixed dose of 1382 Gy and *Canton S* strain as a test case. In preliminary experiments we found that male response was less variable than female response, hence we used males only for our study.

To examine the suitability of our radioassay for the DGRP lines, we selected a panel of 10 DGRP lines at random as well as 3 common *Drosophila* lab strains with distinct genetic backgrounds (*Canton S, yw* and *w^1118^*). The variation in the DGRP lines covered almost the entire spectrum of radiotoxicity (0–95%) with very low standard deviation, while the observed variation in the lab strains ranged from 2–28% (Fig. S1 in File S1).

**Figure 2:**
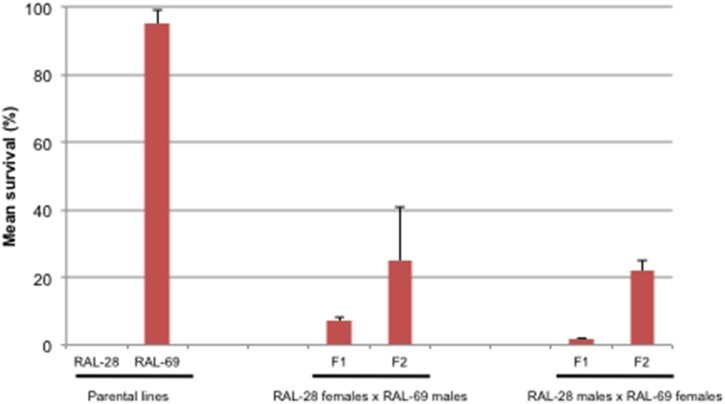
DGRP radioresistance is heritable. Reciprocal crosses between a completely sensitive RAL-28 and a highly resistant RAL-69 lines were set up to generate F1, which were then selfed to produce F2. 50 males from F1 and F2 of each cross were scored for survival after 1382 Gy irradiation. The data shown represents the mean of two independent trials.

We selected two DGRP lines with extreme phenotypes from our pilot panel, RAL-69 and RAL-28, set up reciprocal crosses and scored the radiotoxicity of F1 and F2 males. The parental strains exhibited a similar radiation response as before (0% survival for RAL-28, 95% survival for RAL-69; Fig. 2). The F1s from both crosses showed near-sensitivity of radiation response, while the F2s from both crosses exhibited at least three-fold higher survival than the F1s (*p* = 8.06×10^−3^ by ANOVA). Fitting the parental strains and F1s’ survival to different genetic models identified the most parsimonious model to be a recessive model for radioresistance (Table S4.1). The proportions of survival in F2s were 0.36, 0.14, 0.20, and 0.24, which is close to a recessive proportion of 0.25. Therefore, the results suggest a recessive mode of inheritance for radioresistance for line RAL-69.

Because our assay scored survivors 24 h post-irradiation and since animals that were alive at the 24 h endpoint showed normal life spans, we asked whether the assay was simply finding some strains that took longer to die. To address this, we irradiated two highly resistant strains, RAL-91 and RAL-142, and scored the survivors for up to 10 d (Fig. S2 in File S1). After 10 d, RAL-142 showed 93% survival, and RAL-91 70%. Also, the flies were normal in their behavior and no visible mutations were observed.

We then screened the remaining DGRP lines for survival 24 h post-irradiation, and found extensive phenotypic and genetic variation in radiation response (Figure 1, Table S2). We found that the lines fell into two distinct groups: one group of 92 lines with no survivors at all, another group of 62 lines with survivors. We designated the former group as sensitive and the latter as resistant. Among the resistant group, there was extensive phenotypic variation (ranging from 1% to 98%, Table S2) and a subset of lines were highly variable (coefficient of variation >25%). To test the stability of the radiation resistance phenotype, we selected 12 highly resistant lines and retested them after a period of five to fourteen months (Table S5). The Spearman’s rank correlation coefficient between the two measures was 0.56 with a marginally significant p-value of 0.06. The measures in the second test were systemically lower than those in the first test for unclear reasons (*p* = 4.78×10^−3^ by a paired t-test); however, all the lines maintained their radioresistance. For the resistant lines that were highly variable (RAL-149, -237 and -378), we performed up to 7 replications for 12 lines but they still remained variable (data not shown). We speculated that these lines were inherently variable.

The DGRP lines vary in *Wolbachia* infection status [15], but this parameter had no significant effect on the overall DGRP radiation response (Tables S3 and S6).

### SNPs associated with radiation resistance

To identify genes that harbor alleles conferring inter-individual differences in radiation resistance, we performed a case-control GWA analysis of the phenotype using SNPs from the DGRP freeze 1 sequencing data [15]. Since our extreme phenotype line crosses demonstrated that radioresistance is a recessive trait (Fig. 2), we defined resistant lines as cases and sensitive ones as controls. There were 3,030,570 SNPs that met the quality control thresholds were tested for association with radioresistance using a linear mixed model.

**Figure 3:**
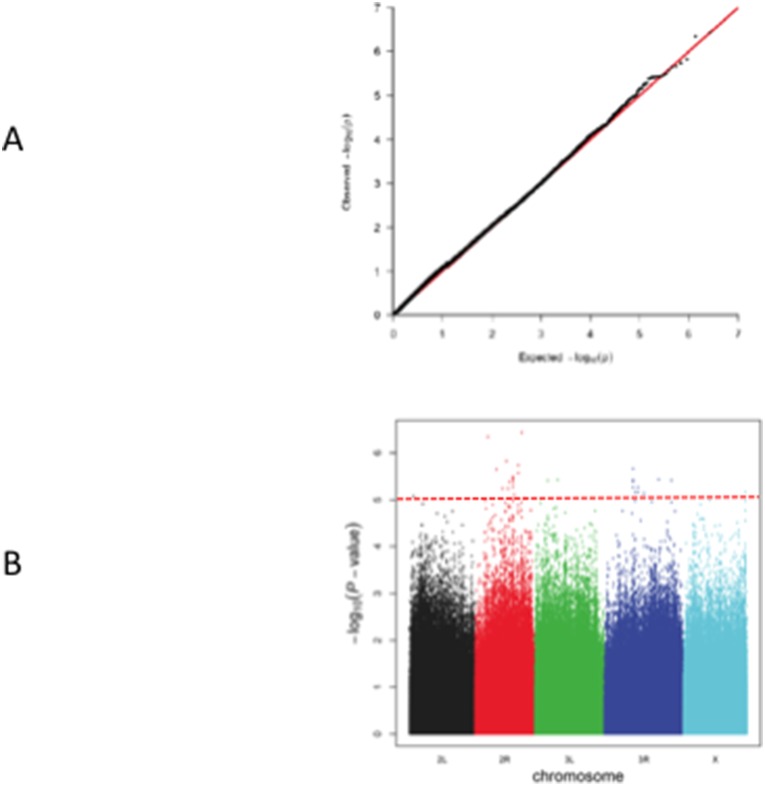
Association analyses of radiation resistance among 154 DGRP lines. (A) Quantile-quantile plot. The red line indicates the expected and the black line the observed *p* values. (B) Manhattan plot of *p* values. The red dashed line indicates *p*<10^−5^.

The quantile-quantile plot of *p* values showed no systematic deviation from the null distribution (Fig. 3A). A total of 334,729 LD blocks were defined, which led to a genome-wide significance threshold of 1.49×10^−7^. The lowest *p* value in our screen was 3.66×10^−7^, with none of the SNPs exceeding the genome-wide significance threshold. We estimated the sample size needed to exceed *p* = 1.49×10^−7^ with a power of 0.8 for the top 32 SNPs, and found the required sample size to range from 265 (for 3R_10180595) to 793 (for 2R_13959724). As the DGRP consists of lines derived from a natural population, the prevalence of radioresistance was set to be 0.4 (62/154). The heritability using the whole-genome variation was estimated to be 0.067; in contrast, by linear mixed models accounting for line effects, the heritability was estimated to be >0.8 (Table S4).

**Figure 4:**
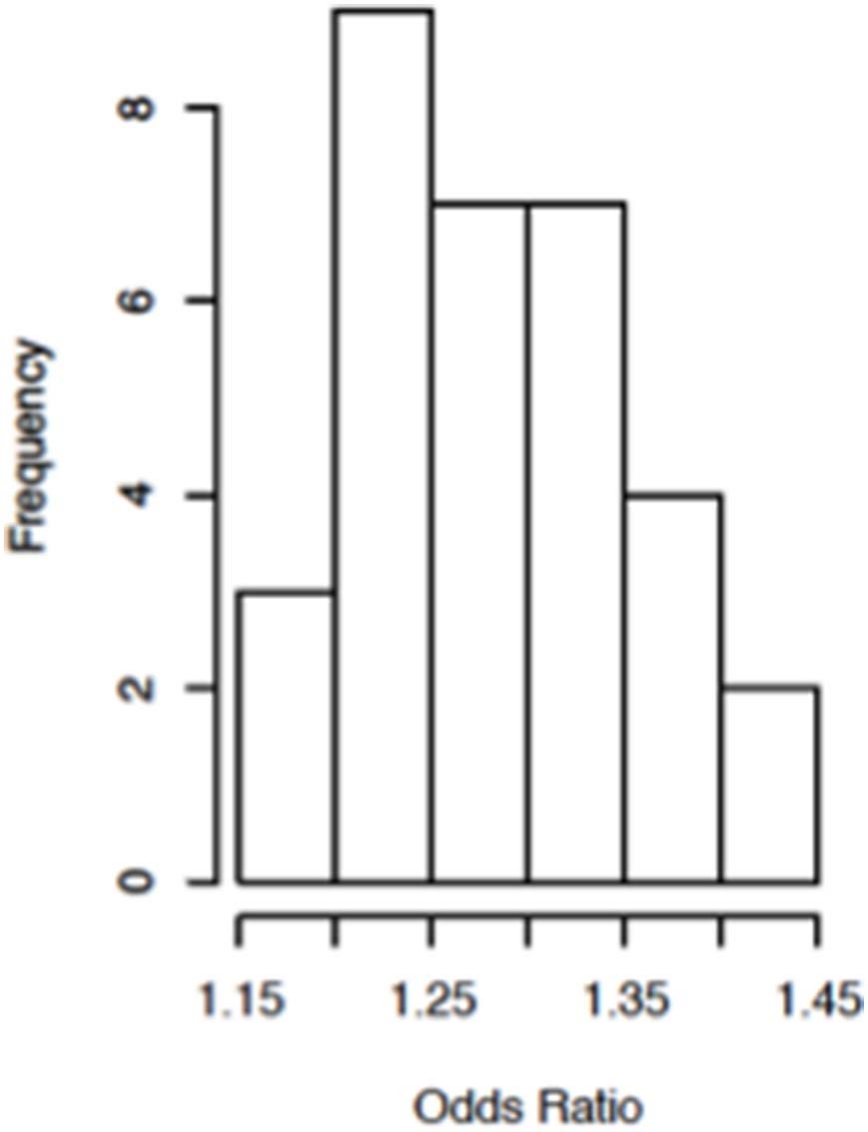
Odds ratio histogram of top 32 SNPs at p<10^−5^.

A total of 32 SNPs were associated with radiation resistance at *p*<10^−5^, among which two were at *p*<10^−6^ (Table S7; Fig. 3B). The majority of the 32 SNPs were common variants, having minor allele frequency of at least 0.14. Their effect sizes (odds ratios) were similar, ranging from 1.19 to 1.44 (Fig. 4).

According to site class, of the 32 SNPs, 15 were intronic, 8 fell in coding regions (two non-synonymous, 6 synonymous), two occurred in 3′ UTR, 6 were intergenic and one was located in a gene desert. Excluding the one in the gene desert, the remaining SNPs implicated 24 genes, 9 of which have human homologs. All 24 genes are novel candidates in radiation resistance (Table S8). Pathway analysis using the DAVID software (http://david.abcc.ncifcrf.gov/) showed that the 32 SNPs were not enriched in any known biological process or pathways involved in radiation resistance. Furthermore, the 9 human homologs also were not enriched in any known biological pathways.

**Figure 5:**
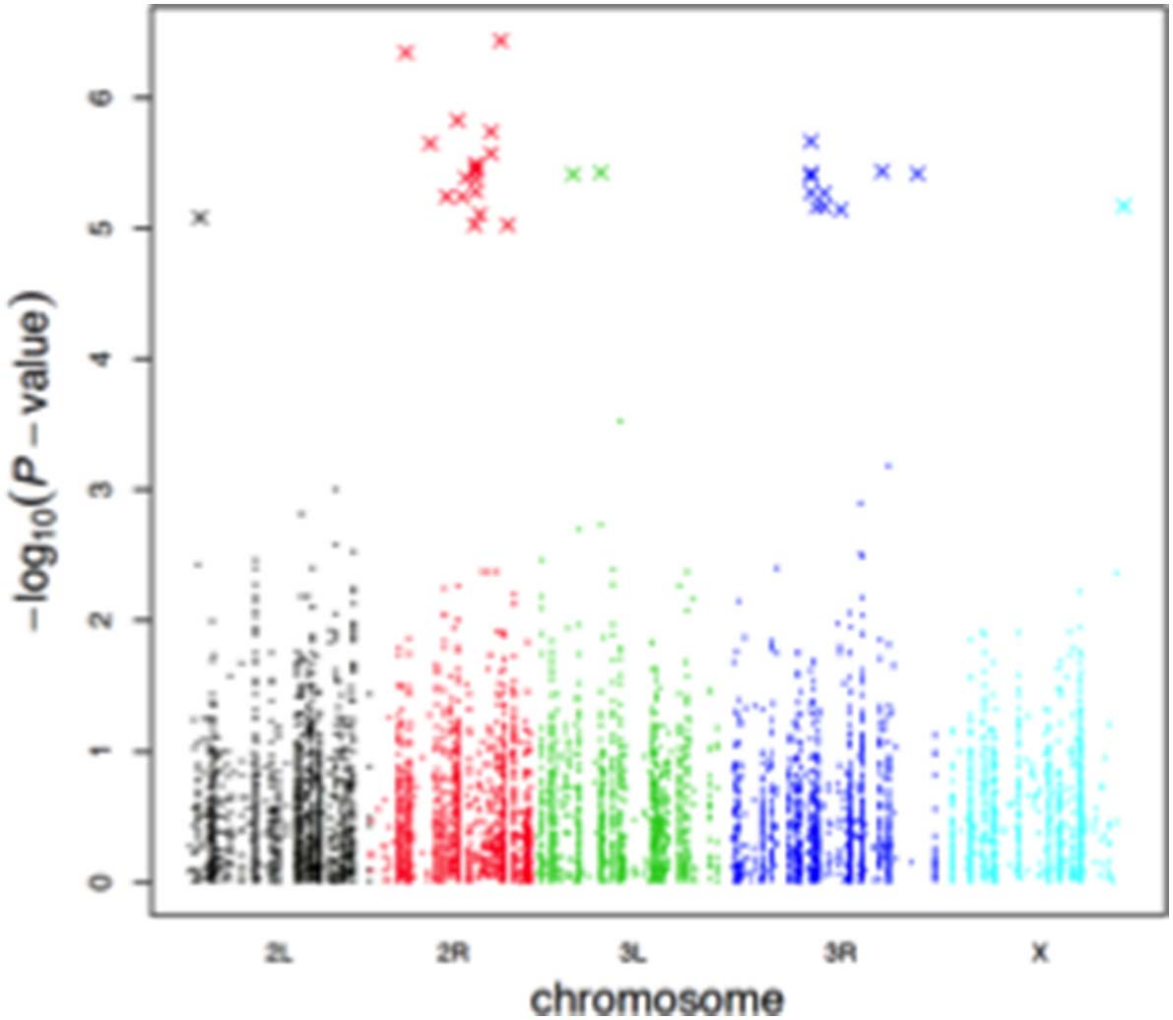
Variants in DNA damage and repair genes are not among the top associated SNPs. A scatterplot of *p* values for all 10,916 SNPs representing a comprehensive set of 102 DNA damage and repair genes (dots), along with the *p* values of top 32 SNPs (crosses).

To determine whether canonical DNA damage and repair gene variants were present or enriched among the significant hits in our screen, we considered a comprehensive list of 102 genes involved in DNA damage response, of which 97 had human homologs, based on the compilation done by Wood et al. and Lange et al. [24], [25], [26]. The DGRP set harbored a total of 10,916 variants (excluding intergenic SNPs) in these genes (Table S9). We found that none of the DNA damage and repair gene variants were present among the significant SNPs (Fig. 5). Table S10 lists the lowest *p* value variant of each of the 102 DNA damage and repair genes.

## Discussion

Genes involved in radiation toxicity/resistance have largely been identified using either cultured cells or laboratory-engineered single gene mutation studies in animal models. GWA studies have emerged as a powerful approach to circumvent the limitations of cultured cells and single gene mutation studies, uncovering novel loci involved in a number of complex traits that play a role in whole organism [20]. We therefore leveraged the publicly available DGRP resource harboring natural variation in *Drosophila* to discover novel variants involved in radiation resistance by using a whole animal radioassay. Although ionizing radiation is not expected to be strong in contemporaneous environments, and is not an important agent of natural selection in the wild, it is an important consideration for manned space expeditions.

This is the first initiative to approach determinants of radiation resistance in whole animals. As a heritable and complex trait, post-irradiation survival is clearly an intriguing phenotype. We considered several experimental phenotypes with aim to devise an experimental design in which feasibility was a pivotal consideration. For example, we counted the number of offsprings of radiation resistant and sensitive flies as an endpoint, and found no significant difference (data not shown). Next, we considered establishing the LD50 values for all 192 strains but quickly found that this would not be practical. We also considered larval irraidation, where the endpoint would be larval survival or death; however, it is well documented that different strains can differ with respect to developmental pace. Furthermore, radiation sensitivity is highly variable at different stages in development. Therefore, by challenging mature adults we were able to focus the study on our trait of interest (radio-toxicity) without introducing confounding variation derived from differences in developmental rates.

Our screen is the largest effort to date utilizing the natural variation in a model organism to examine the genetic basis of radiation resistance as a complex trait. Our phenotype was heritable and robust, measuring true survival rather than variations in time-to-death (Fig. 2 and S2). For practical reasons, our protocol included just two replicates per line. Therefore, heritability estimation using traditional linear mixed from this data are severely limited. We speculate the line effects and random errors are not identifiable from each other given two replicates per line. The minimal heritability estimate from the whole-genome variation was consistent with the GWA screen results of lacking significant signals; it might suggest a bulk of non-additive genetic effects on radioresistance [27].

We discovered 24 novel *Drosophila* genes (of which 9 have human homologs), encompassing a total of 31 SNPs, likely to be involved in radiation resistance, as well as a gene desert SNP that might play a regulatory role. None of the 10,916 variants in 102 canonical DNA damage and repair genes were present among the 32 significant hits. This finding is consistent with the failure of 49 candidate-gene studies covering 3,144 SNPs in 1,494 human genes to discover variants associated with radiation sensitivity [10], while only 3 human GWA studies on radiotoxicity have identified variants in two novel genes, *FSHR* and *PRDM1* [12]. Therefore, our results support the hypothesis that genetic determinants of whole animal radio-toxicity could radically differ from determinants accessed through cultured cells or single gene mutant animal studies.

Among the genes represented by the significant SNP hits, four contain multiple SNPs (*NK7.1, lack, pnr, CG14621*). Future studies will aim to ascertain whether, biologically, they have been truly selected or are simply in linkage disequilibrium with causative variation in other loci. The genes we identified are not within the QTL regions Q1, Q2, Q3, MT1 and MT2 identified by Gomez et al [28].

SNPs identified in human GWA studies tend to have a modest effect size ranging from 1 to 3, with the majority having odds ratios in the range of 1 to 1.1 [29]. The effect sizes of significant hits in our screen were also modest, ranging from 1.19 to 1.44. Our results demonstrate that even an inbred, model system such as the DGRP, in which each loci is homozygosed and no SNP is left unsequenced, does not provide us with the required sensitivity and specificity for individualized prognosis. This suggests that the goal of personalized medicine is very, very distant – if achievable at all – and that precision medicine should be the aim of future GWA studies [29], [30].

Our screen did not yield any SNP that passed the genome-wide significance threshold of *p* value<1.49×10^−7^ based on the LD structure of the *Drosophila* genome, suggesting that the DGRP resource is underpowered for this trait. We would need a minimum sample size of 265 for at least one among the 32 significant hits and a maximum of 793 lines for all 32 SNPs to pass the genome-wide significance threshold. However, the DGRP resource is adequately powered for complex traits such as oxidative stress resistance and tunicamycin-induced ER stress resistance [31], [32]. This suggests that certain complex traits, such as radiation resistance, require higher DGRP sample size than is available. An important objective for future complex trait research is to anticipate features of dichotomous traits under study that will allow us to predict power requirements early on in the study, prior to a full-scale phenotypic analysis.

It is highly unlikely that ionizing radiation was a source of selective pressure during fly evolution and, therefore, we do not propose that our work is accessing a ‘defense against radiation’. We stress that the same holds true for patients exposed to this same stressor in the clinic, despite differential sensitivities that are, in part, genetic. Like most gene-directed differences in perturbation responses, ionizing radiation is probably a surrogate for other environmental challenges that mold complex traits. In fact, an important motivation for this study was to extract informative clues that could advance our understanding of the underlying radiation biology. We have not investigated radiation-stable proteins in the present study and certainly it is possible that protein stability plays a part in radiation response. Whatever the ultimate mechanism turns out to be, the underlying architecture governing these differences are distinct when accessed in whole animals versus cultured cells.

## Supporting Information

Table S1 Association between radioresistance and the top five principal components derived from the whole genome SNP data of 154 DGRP lines.
(DOCX)

Table S2 Raw and mean survival values of 154 DGRP lines.
(DOCX)

Table S3 *Wolbachia* infection status and mean radiation response values of 154 DGRP lines.
(DOCX)

Table S4 Heritability estimation of radioresistance. **Table S4.1**. Inferring the genetic model of radioresistance by reciprocal cross of the sensitive line RAL-28 and the resistant line RAL-69.
(DOCX)

Table S5 Temporal phenotypic stability study of 12 highly resistant DGRP lines.
(DOCX)

Table S6 *Wolbachia* infection status has no signficant effect on the overall DGRP radiation response.
(DOCX)

Table S7 GWA analysis results.
(XLSX)

Table S8 Functional annotation of top candidate genes.
(XLSX)

Table S9 Number of SNPs and human homologs of *Drosophila* DNA damage and repair genes.
(XLSX)

Table S10 Lowest *p* value SNP in a select list of DNA damage and repair genes.
(XLSX)

File S1 Contains the following files: **Figure S1.** Variation in radiation response among common *Drosophila* lab strains and 10 DGRP lines. The lab strains used were *Canton S, yw* and *w^1118^*. The 10 DGRP lines were chosen at random. 50 males from each line were aged for 7–11 days, irradiated at 1382 Gy and the number of survivors were scored 24 hours post-irradiation. The data shown represents the mean of two independent trials. **Figure S2.** Highly resistant DGRP strains survive over a long period following irradiation. 50 males from RAL-91 and RAL-142 strains were aged for 7–11 days, irradiated with 1382 Gy and scored for survivors over a period of 10 days.
(PPT)

## References

[1] Mettler FA Jr, Voelz GL (2002) Major radiation exposure–what to expect and how to respond. N Engl J Med 346: 1554–1561. 12015396 10.1056/NEJMra000365

[2] Suzuki K, Ojima M, Kodama S, Watanabe M (2003) Radiation-induced DNA damage and delayed induced genomic instability. Oncogene 22: 6988–6993. 14557802 10.1038/sj.onc.1206881

[3] Morgan WF (2003) Non-targeted and delayed effects of exposure to ionizing radiation: I. Radiation-induced genomic instability and bystander effects in vitro. Radiat Res 159: 567–580. 12710868 10.1667/0033-7587(2003)159[0567:nadeoe]2.0.co;2

[4] Little MP (2003) Risks associated with ionizing radiation. Br Med Bull 68: 259–275. 14757722 10.1093/bmb/ldg031

[5] Boice JD Jr (2012) Radiation epidemiology: a perspective on Fukushima. J Radiol Prot 32: N33–40. 22395193 10.1088/0952-4746/32/1/N33

[6] Sankaranarayanan K (2006) Estimation of the genetic risks of exposure to ionizing radiation in humans: current status and emerging perspectives. J Radiat Res 47 Suppl B: B57–66. 10.1269/jrr.47.b57 17019053

[7] Finnon P, Robertson N, Dziwura S, Raffy C, Zhang W, et al (2008) Evidence for significant heritability of apoptotic and cell cycle responses to ionising radiation. Hum Genet 123: 485–493. 18437427 10.1007/s00439-008-0500-1

[8] Curwen GB, Cadwell KK, Winther JF, Tawn EJ, Rees GS, et al (2010) The heritability of G2 chromosomal radiosensitivity and its association with cancer in Danish cancer survivors and their offspring. Int J Radiat Biol 86: 986–995. 20807177 10.3109/09553002.2010.496027 PMC3777830

[9] Turesson I, Nyman J, Holmberg E, Oden A (1996) Prognostic factors for acute and late skin reactions in radiotherapy patients. Int J Radiat Oncol Biol Phys 36: 1065–1075. 8985028 10.1016/s0360-3016(96)00426-9

[10] West CM, Dunning AM, Rosenstein BS (2012) Genome-wide association studies and prediction of normal tissue toxicity. Semin Radiat Oncol 22: 91–99. 22385916 10.1016/j.semradonc.2011.12.007

[11] Barnett GC, West CM, Dunning AM, Elliott RM, Coles CE, et al (2009) Normal tissue reactions to radiotherapy: towards tailoring treatment dose by genotype. Nat Rev Cancer 9: 134–142. 19148183 10.1038/nrc2587 PMC2670578

[12] Kerns SL, Ostrer H, Stock R, Li W, Moore J, et al (2010) Genome-wide association study to identify single nucleotide polymorphisms (SNPs) associated with the development of erectile dysfunction in African-American men after radiotherapy for prostate cancer. Int J Radiat Oncol Biol Phys 78: 1292–1300. 20932654 10.1016/j.ijrobp.2010.07.036 PMC2991431

[13] Spencer CC, Su Z, Donnelly P, Marchini J (2009) Designing genome-wide association studies: sample size, power, imputation, and the choice of genotyping chip. PLoS Genet 5: e1000477. 19492015 10.1371/journal.pgen.1000477 PMC2688469

[14] Marian AJ (2012) Molecular genetic studies of complex phenotypes. Transl Res 159: 64–79. 22243791 10.1016/j.trsl.2011.08.001 PMC3259530

[15] Mackay TF, Richards S, Stone EA, Barbadilla A, Ayroles JF, et al (2012) The Drosophila melanogaster Genetic Reference Panel. Nature 482: 173–178. 22318601 10.1038/nature10811 PMC3683990

[16] Mackay T, Richards S, Weinstock G, Gibbs R (2008) Proposal to Sequence a Drosophila Genetic Reference Panel: A Community Resource for the Study of Genotypic and Phenotypic Variation. http://flybaseorg/static_pages/news/whitepapers/Drosophila_Genetic_Reference_Panel_Whitepaperpdf.

[17] Yang J, Lee SH, Goddard ME, Visscher PM (2011) GCTA: A tool for genome-wide complex trait analysis. Am J Hum Genet 88: 76–82. 21167468 10.1016/j.ajhg.2010.11.011 PMC3014363

[18] Price AL, Zaitlen NA, Reich D, Patterson N (2010) New approaches to population stratification in genome-wide association studies. Nat Rev Genet 11: 459–463. 20548291 10.1038/nrg2813 PMC2975875

[19] Xing G, Lin CY, Wooding SP, Xing C (2012) Blindly using Wald’s test can miss rare disease-causal variants in case-control association studies. Ann Hum Genet 76: 168–77. 22256951 10.1111/j.1469-1809.2011.00700.x

[20] Zhou X, Stephens M (2012) Genome-wide efficient mixed-model analysis for association studies. Nat Genet 44: 821–4. 22706312 10.1038/ng.2310 PMC3386377

[21] Gabriel SH, Schaffner SF, Nguyen H, Moore JH, Roy J, et al (2002) The structure of haplotype blocks in the human genome. Science 296: 2225–9. 12029063 10.1126/science.1069424

[22] Purcell S, Neale B, Todd-Brown K, Thomas L, Ferreira MA, et al (2007) PLINK: a tool set for whole-genome association and population-based linkage analysis. Am J Hum Genet 81: 559–75. 17701901 10.1086/519795 PMC1950838

[23] Lee SH, Wray NR, Goddard ME, Visscher PM (2011) Estimating missing heritability for disease from genome-wide association studies. Am J Hum Genet 88: 294–305. 21376301 10.1016/j.ajhg.2011.02.002 PMC3059431

[24] Wood RD, Mitchell M, Lindahl T (2005) Human DNA repair genes, 2005. Mutat Res 577: 275–283. 15922366 10.1016/j.mrfmmm.2005.03.007

[25] Wood RD, Mitchell M, Sgouros J, Lindahl T (2001) Human DNA repair genes. Science 291: 1284–1289. 11181991 10.1126/science.1056154

[26] Lange SS, Takata K, Wood RD (2011) DNA polymerases and cancer. Nat Rev Cancer 11: 96–110. 21258395 10.1038/nrc2998 PMC3739438

[27] Yang J, Benyamin B, McEvoy BP, Gordon S, Henders AK, et al (2010) Common SNPs explain a large proportion of the heritability for human height. Nat Genet 42: 565–569. 20562875 10.1038/ng.608 PMC3232052

[28] Gomez FH, Loeschcke V, Norry FM (2013) QTL for survival to UV-C radiation in Drosophila melanogaster. Int J Radiat Biol. 89: 583–589. 10.3109/09553002.2012.711503 22788381

[29] Pawitan Y, Seng KC, Magnusson PK (2009) How many genetic variants remain to be discovered? PLoS One 4: e7969. 19956539 10.1371/journal.pone.0007969 PMC2780697

[30] Katsnelson A (2013) Momentum grows to make ‘personalized’ medicine more ‘precise’. Nat Med 19: 249. 23467220 10.1038/nm0313-249

[31] Weber AL, Khan GF, Magwire MM, Tabor CL, Mackay TF, et al (2012) Genome-wide association analysis of oxidative stress resistance in Drosophila melanogaster. PLoS One 7: e34745. 22496853 10.1371/journal.pone.0034745 PMC3319608

[32] Chow CY, Wolfner MF, Clark AG (2013) Using natural variation in Drosophila to discover previously unknown endoplasmic reticulum stress genes. Proc Natl Acad Sci USA 110: 9013–9018. 23667151 10.1073/pnas.1307125110 PMC3670321

