# Supplementary material for: Genome-Wide Association Analysis of Radiation Resistance in Drosophila melanogaster
Source: PLoS One. 2014 Aug 14;9(8):e104858. doi: 10.1371/journal.pone.0104858 (PMC4133248; doi:10.1371/journal.pone.0104858)
Supplement: Table S1 — Association between radioresistance and the top five principal components derived from the whole genome SNP data of 154 DGRP lines. (DOCX) [file pone.0104858.s001.docx]

**Table S1.** Association* between radio resistance and the top five principal components derived from the whole-genome SNP data for the 154 DGRP lines.

| Principal Components | Estimate of coefficient | Standard deviation | *p*-value |
| --- | --- | --- | --- |
| 1 | 2.37 | 2.26 | 0.27 |
| 2 | -3.09 | 2.15 | 0.15 |
| 3 | -2.81 | 2.24 | 0.21 |
| 4 | 0.62 | 2.13 | 0.77 |
| 5 | -0.60 | 2.02 | 0.77 |

*A logistic regression model was employed to model the association between radio resistance and each principal component.
