# Supplementary material for: Genome-Wide Association Analysis of Radiation Resistance in Drosophila melanogaster
Source: PLoS One. 2014 Aug 14;9(8):e104858. doi: 10.1371/journal.pone.0104858 (PMC4133248; doi:10.1371/journal.pone.0104858)
Supplement: Table S2 — Raw and mean survival values of 154 DGRP lines. (DOCX) [file pone.0104858.s002.docx]

**Table S2.** Raw and mean survival values of 154 DGRP lines.

SD = standard deviation

CV = coefficient of variation

NA = not applicable

| **RAL #** | **Trial 1 raw** | **Trial 2 raw** | **Mean %** | **SD** | **CV** |
| --- | --- | --- | --- | --- | --- |
| 21 | 0 | 0 | 0 | 0.00 | NA |
| 26 | 0 | 0 | 0 | 0.00 | NA |
| 28 | 0 | 0 | 0 | 0.00 | NA |
| 38 | 4 | 0 | 4 | 5.66 | 141.42 |
| 40 | 0 | 0 | 0 | 0.00 | NA |
| 41 | 0 | 0 | 0 | 0.00 | NA |
| 42 | 11 | 9 | 20 | 2.83 | 14.14 |
| 45 | 0 | 0 | 0 | 0.00 | NA |
| 57 | 48 | 38 | 86 | 14.14 | 16.44 |
| 59 | 11 | 9 | 20 | 2.83 | 14.14 |
| 69 | 46 | 49 | 95 | 4.24 | 4.47 |
| 73 | 11 | 34 | 45 | 32.53 | 72.28 |
| 75 | 6 | 5 | 11 | 1.41 | 12.86 |
| 83 | 18 | 16 | 34 | 2.83 | 8.32 |
| 85 | 7 | 10 | 17 | 4.24 | 24.96 |
| 88 | 12 | 41 | 53 | 41.01 | 77.38 |
| 91 | 50 | 48 | 98 | 2.83 | 2.89 |
| 93 | 15 | 13 | 28 | 2.83 | 10.10 |
| 101 | 0 | 0 | 0 | 0.00 | NA |
| 105 | 0 | 4 | 4 | 5.66 | 141.42 |
| 109 | 0 | 0 | 0 | 0.00 | NA |
| 129 | 13 | 15 | 28 | 2.83 | 10.10 |
| 136 | 0 | 0 | 0 | 0.00 | NA |
| 138 | 0 | 0 | 0 | 0.00 | NA |
| 142 | 34 | 48 | 82 | 19.80 | 24.15 |
| 149 | 25 | 10 | 35 | 21.21 | 60.61 |
| 153 | 0 | 0 | 0 | 0.00 | NA |
| 158 | 0 | 0 | 0 | 0.00 | NA |
| 161 | 0 | 0 | 0 | 0.00 | NA |
| 176 | 0 | 0 | 0 | 0.00 | NA |
| 177 | 0 | 0 | 0 | 0.00 | NA |
| 181 | 10 | 7 | 17 | 4.24 | 24.96 |
| 195 | 2 | 2 | 4 | 0 | 0 |
| 208 | 40 | 49 | 89 | 12.73 | 14.30 |
| 217 | 0 | 0 | 0 | 0.00 | NA |
| 227 | 0 | 0 | 0 | 0.00 | NA |
| 228 | 0 | 0 | 0 | 0.00 | NA |
| 229 | 7 | 9 | 16 | 2.83 | 17.68 |
| 233 | 0 | 0 | 0 | 0.00 | NA |
| 235 | 0 | 0 | 0 | 0.00 | NA |
| 237 | 17 | 40 | 57 | 32.53 | 57.06 |
| 239 | 0 | 0 | 0 | 0.00 | NA |
| 256 | 10 | 7 | 17 | 4.24 | 24.96 |
| 272 | 0 | 8 | 8 | 11.31 | 141.42 |
| 280 | 0 | 0 | 0 | 0.00 | NA |
| 287 | 3 | 11 | 14 | 11.31 | 80.81 |
| 301 | 0 | 0 | 0 | 0.00 | NA |
| 303 | 0 | 0 | 0 | 0.00 | NA |
| 304 | 4 | 4 | 8 | 0.00 | 0.00 |
| 309 | 0 | 0 | 0 | 0.00 | NA |
| 310 | 0 | 0 | 0 | 0.00 | NA |
| 313 | 0 | 0 | 0 | 0.00 | NA |
| 317 | 0 | 0 | 0 | 0.00 | NA |
| 318 | 23 | 26 | 49 | 4.24 | 8.66 |
| 320 | 0 | 0 | 0 | 0.00 | NA |
| 321 | 4 | 3 | 7 | 1.41 | 20.20 |
| 325 | 1 | 1 | 2 | 0 | 0 |
| 332 | 2 | 2 | 4 | 0 | 0 |
| 338 | 50 | 40 | 90 | 14.14 | 15.71 |
| 350 | 0 | 0 | 0 | 0.00 | NA |
| 352 | 0 | 0 | 0 | 0.00 | NA |
| 356 | 0 | 0 | 0 | 0.00 | NA |
| 357 | 2 | 1 | 3 | 1.41 | 47.14 |
| 358 | 0 | 0 | 0 | 0.00 | NA |
| 359 | 0 | 0 | 0 | 0.00 | NA |
| 362 | 0 | 0 | 0 | 0.00 | NA |
| 365 | 0 | 0 | 0 | 0.00 | NA |
| 367 | 12 | 14 | 26 | 2.83 | 10.88 |
| 370 | 36 | 30 | 66 | 8.49 | 12.86 |
| 371 | 0 | 0 | 0 | 0.00 | NA |
| 373 | 0 | 6 | 6 | 8.49 | 141.42 |
| 374 | 5 | 1 | 6 | 5.66 | 94.28 |
| 375 | 13 | 10 | 23 | 4.24 | 18.45 |
| 377 | 0 | 0 | 0 | 0.00 | NA |
| 378 | 38 | 18 | 56 | 28.28 | 50.51 |
| 379 | 0 | 0 | 0 | 0.00 | NA |
| 380 | 15 | 0 | 15 | 21.21 | 141.42 |
| 381 | 0 | 0 | 0 | 0.00 | NA |
| 383 | 0 | 0 | 0 | 0.00 | NA |
| 386 | 0 | 0 | 0 | 0.00 | NA |
| 391 | 0 | 0 | 0 | 0.00 | NA |
| 392 | 4 | 5 | 9 | 1.41 | 15.71 |
| 398 | 4 | 4 | 8 | 0 | 0 |
| 399 | 0 | 0 | 0 | 0.00 | NA |
| 405 | 24 | 28 | 52 | 5.66 | 10.88 |
| 406 | 0 | 0 | 0 | 0.00 | NA |
| 409 | 2 | 3 | 5 | 1.41 | 28.28 |
| 426 | 0 | 0 | 0 | 0.00 | NA |
| 427 | 0 | 0 | 0 | 0.00 | NA |
| 437 | 0 | 0 | 0 | 0.00 | NA |
| 439 | 0 | 0 | 0 | 0.00 | NA |
| 440 | 0 | 0 | 0 | 0.00 | NA |
| 443 | 0 | 0 | 0 | 0.00 | NA |
| 461 | 0 | 0 | 0 | 0.00 | NA |
| 491 | 0 | 0 | 0 | 0.00 | NA |
| 492 | 47 | 37 | 84 | 14.14 | 16.84 |
| 502 | 5 | 6 | 11 | 1.41 | 12.86 |
| 508 | 1 | 1 | 2 | 0 | 0 |
| 509 | 0 | 0 | 0 | 0.00 | NA |
| 513 | 0 | 0 | 0 | 0.00 | NA |
| 517 | 0 | 0 | 0 | 0.00 | NA |
| 531 | 0 | 0 | 0 | 0.00 | NA |
| 535 | 14 | 18 | 32 | 5.66 | 17.68 |
| 554 | 0 | 0 | 0 | 0.00 | NA |
| 555 | 0 | 0 | 0 | 0.00 | NA |
| 563 | 0 | 0 | 0 | 0.00 | NA |
| 589 | 0 | 0 | 0 | 0.00 | NA |
| 595 | 13 | 12 | 25 | 1.41 | 5.66 |
| 639 | 0 | 0 | 0 | 0.00 | NA |
| 642 | 0 | 0 | 0 | 0.00 | NA |
| 646 | 10 | 9 | 19 | 1.41 | 7.44 |
| 703 | 0 | 0 | 0 | 0.00 | NA |
| 705 | 6 | 5 | 11 | 1.41 | 12.86 |
| 707 | 0 | 0 | 0 | 0.00 | NA |
| 712 | 0 | 0 | 0 | 0.00 | NA |
| 714 | 0 | 0 | 0 | 0.00 | NA |
| 716 | 7 | 6 | 13 | 1.41 | 10.88 |
| 721 | 0 | 0 | 0 | 0.00 | NA |
| 727 | 8 | 13 | 21 | 7.07 | 33.67 |
| 730 | 0 | 0 | 0 | 0.00 | NA |
| 732 | 0 | 0 | 0 | 0.00 | NA |
| 737 | 0 | 0 | 0 | 0.00 | NA |
| 738 | 0 | 0 | 0 | 0.00 | NA |
| 757 | 0 | 0 | 0 | 0.00 | NA |
| 761 | 0 | 0 | 0 | 0.00 | NA |
| 765 | 0 | 0 | 0 | 0.00 | NA |
| 774 | 18 | 2 | 20 | 22.63 | 113.14 |
| 776 | 2 | 2 | 4 | 0 | 0 |
| 783 | 2 | 28 | 30 | 36.77 | 122.57 |
| 786 | 1 | 1 | 2 | 0 | 0 |
| 787 | 0 | 0 | 0 | 0.00 | NA |
| 790 | 0 | 0 | 0 | 0.00 | NA |
| 796 | 0 | 0 | 0 | 0.00 | NA |
| 799 | 0 | 0 | 0 | 0.00 | NA |
| 801 | 0 | 9 | 9 | 12.73 | 141.42 |
| 802 | 0 | 0 | 0 | 0.00 | NA |
| 804 | 0 | 1 | 1 | 1.41 | 141.42 |
| 805 | 0 | 0 | 0 | 0.00 | NA |
| 808 | 35 | 28 | 63 | 9.90 | 15.71 |
| 810 | 0 | 0 | 0 | 0.00 | NA |
| 812 | 0 | 5 | 5 | 7.07 | 141.42 |
| 818 | 1 | 13 | 14 | 16.97 | 121.22 |
| 820 | 0 | 0 | 0 | 0.00 | NA |
| 822 | 0 | 0 | 0 | 0.00 | NA |
| 832 | 0 | 0 | 0 | 0.00 | NA |
| 837 | 0 | 0 | 0 | 0.00 | NA |
| 852 | 0 | 0 | 0 | 0.00 | NA |
| 855 | 24 | 10 | 34 | 19.80 | 58.23 |
| 857 | 7 | 7 | 14 | 0 | 0 |
| 859 | 0 | 0 | 0 | 0.00 | NA |
| 861 | 0 | 0 | 0 | 0.00 | NA |
| 879 | 38 | 40 | 78 | 2.83 | 3.63 |
| 882 | 0 | 0 | 0 | 0.00 | NA |
| 887 | 0 | 0 | 0 | 0.00 | NA |
| 890 | 0 | 0 | 0 | 0.00 | NA |
| 892 | 0 | 1 | 1 | 1.41 | 141.42 |
| 894 | 0 | 5 | 5 | 7.07 | 141.42 |
| 897 | 0 | 0 | 0 | 0.00 | NA |
| 907 | 8 | 0 | 8 | 11.31 | 141.42 |
| 908 | 0 | 0 | 0 | 0.00 | NA |
| 911 | 0 | 0 | 0 | 0.00 | NA |
