# Supplementary material for: Genome-Wide Association Analysis of Radiation Resistance in Drosophila melanogaster
Source: PLoS One. 2014 Aug 14;9(8):e104858. doi: 10.1371/journal.pone.0104858 (PMC4133248; doi:10.1371/journal.pone.0104858)
Supplement: Table S3 — Wolbachia infection status and mean radiation response values of 154 DGRP lines. (DOCX) [file pone.0104858.s003.docx]

**Table S3.** *Wolbachia* infection status (WI) and mean radiation response values of 154 DGRP lines. Note that *Wolbachia* infection status is from Reference 15

| **RAL #** | **Mean %** | **WI** |
| --- | --- | --- |
| 21 | 0 | y |
| 26 | 0 | n |
| 28 | 0 | n |
| 38 | 4 | n |
| 40 | 0 | y |
| 41 | 0 | n |
| 42 | 20 | n |
| 45 | 0 | n |
| 57 | 86 | n |
| 59 | 20 | n |
| 69 | 95 | y |
| 73 | 45 | y |
| 75 | 11 | y |
| 83 | 34 | n |
| 85 | 17 | n |
| 88 | 53 | n |
| 91 | 98 | n |
| 93 | 28 | n |
| 101 | 0 | n |
| 105 | 4 | n |
| 109 | 0 | n |
| 129 | 28 | n |
| 136 | 0 | y |
| 138 | 0 | n |
| 142 | 82 | y |
| 149 | 35 | y |
| 153 | 0 | y |
| 158 | 0 | n |
| 161 | 0 | n |
| 176 | 0 | y |
| 177 | 0 | n |
| 181 | 17 | y |
| 195 | 4 | n |
| 208 | 89 | n |
| 217 | 0 | n |
| 227 | 0 | y |
| 228 | 0 | n |
| 229 | 16 | n |
| 233 | 0 | n |
| 235 | 0 | n |
| 237 | 57 | y |
| 239 | 0 | n |
| 256 | 17 | y |
| 272 | 8 | NA |
| 280 | 0 | y |
| 287 | 14 | y |
| 301 | 0 | ND |
| 303 | 0 | n |
| 304 | 8 | ND |
| 309 | 0 | n |
| 310 | 0 | y |
| 313 | 0 | n |
| 317 | 0 | y |
| 318 | 49 | y |
| 320 | 0 | y |
| 321 | 7 | y |
| 325 | 2 | n |
| 332 | 4 | n |
| 338 | 90 | y |
| 350 | 0 | n |
| 352 | 0 | y |
| 356 | 0 | y |
| 357 | 3 | n |
| 358 | 0 | n |
| 359 | 0 | n |
| 362 | 0 | y |
| 365 | 0 | y |
| 367 | 26 | n |
| 370 | 66 | y |
| 371 | 0 | n |
| 373 | 6 | n |
| 374 | 6 | y |
| 375 | 23 | n |
| 377 | 0 | n |
| 378 | 56 | NA |
| 379 | 0 | n |
| 380 | 15 | y |
| 381 | 0 | n |
| 383 | 0 | y |
| 386 | 0 | n |
| 391 | 0 | n |
| 392 | 9 | n |
| 398 | 8 | NA |
| 399 | 0 | n |
| 405 | 52 | y |
| 406 | 0 | n |
| 409 | 5 | y |
| 426 | 0 | n |
| 427 | 0 | n |
| 437 | 0 | n |
| 439 | 0 | n |
| 440 | 0 | y |
| 443 | 0 | n |
| 461 | 0 | y |
| 491 | 0 | NA |
| 492 | 84 | n |
| 502 | 11 | n |
| 508 | 2 | n |
| 509 | 0 | n |
| 513 | 0 | y |
| 517 | 0 | n |
| 531 | 0 | y |
| 535 | 32 | y |
| 554 | 0 | NA |
| 555 | 0 | y |
| 563 | 0 | n |
| 589 | 0 | y |
| 595 | 25 | y |
| 639 | 0 | y |
| 642 | 0 | n |
| 646 | 19 | y |
| 703 | 0 | n |
| 705 | 11 | y |
| 707 | 0 | y |
| 712 | 0 | y |
| 714 | 0 | n |
| 716 | 13 | y |
| 721 | 0 | y |
| 727 | 21 | y |
| 730 | 0 | y |
| 732 | 0 | n |
| 737 | 0 | y |
| 738 | 0 | y |
| 757 | 0 | n |
| 761 | 0 | y |
| 765 | 0 | n |
| 774 | 20 | n |
| 776 | 4 | y |
| 783 | 30 | y |
| 786 | 2 | y |
| 787 | 0 | y |
| 790 | 0 | y |
| 796 | 0 | y |
| 799 | 0 | y |
| 801 | 9 | y |
| 802 | 0 | y |
| 804 | 1 | y |
| 805 | 0 | y |
| 808 | 63 | n |
| 810 | 0 | n |
| 812 | 5 | n |
| 818 | 14 | y |
| 820 | 0 | y |
| 822 | 0 | y |
| 832 | 0 | y |
| 837 | 0 | y |
| 852 | 0 | y |
| 855 | 34 | y |
| 857 | 14 | n |
| 859 | 0 | y |
| 861 | 0 | y |
| 879 | 78 | y |
| 882 | 0 | y |
| 887 | 0 | y |
| 890 | 0 | y |
| 892 | 1 | y |
| 894 | 5 | n |
| 897 | 0 | y |
| 907 | 8 | n |
| 908 | 0 | n |
| 911 | 0 | n |
