# Supplementary material for: Genome-Wide Association Analysis of Radiation Resistance in Drosophila melanogaster
Source: PLoS One. 2014 Aug 14;9(8):e104858. doi: 10.1371/journal.pone.0104858 (PMC4133248; doi:10.1371/journal.pone.0104858)
Supplement: Table S4 — Heritability estimation of radioresistance. Table S4.1. Inferring the genetic model of radioresistance by reciprocal cross of the sensitive line RAL-28 and the resistant line RAL-69. (DOCX) [file pone.0104858.s004.docx]

**Table S4.** Heritability estimation of radioresistence.

| Method | Source | Variance | Standard Deviation | Heritability |
| --- | --- | --- | --- | --- |
| Genomic profiling* |  | 0.010 | 0.064 | 0.067* |
|  |  | 0.224 | 0.123 |  |
| Linear mixed model I** |  | 116.8 | 10.807 | 0.878 |
|  |  | 16.3 | 4.037 |  |
| Linear mixed model II*** |  | 173.39 | 13.168 | 0.813 |
|  |  | 39.77 | 6.306 |  |

*A liability threshold model based on the whole-genome variation; the heritability was transformed based on a pre-specified prevalence of 0.4.

**A linear mixed model testing for familial / line effects based on all 154 lines.

*** A linear mixed model testing for familial / line effects based on the 92 resistant lines.

**Table S4.1.** Inferring the genetic model of radioresistance by reciprocal cross of the sensitive line RAL-28 and the resistant line RAL-69.

| Genetic Model | Degree of freedom | Log(Likelihood) |
| --- | --- | --- |
| Full model | 4 | -12.7 |
| Recessive Model | 3 | -14.6 |
| Additive Model | 3 | -30.4 |
| Dominant Model | 3 | -34.7 |
