# Supplementary material for: Genome-Wide Association Analysis of Radiation Resistance in Drosophila melanogaster
Source: PLoS One. 2014 Aug 14;9(8):e104858. doi: 10.1371/journal.pone.0104858 (PMC4133248; doi:10.1371/journal.pone.0104858)
Supplement: Table S5 — Temporal phenotypic stability study of 12 highly resistant DGRP lines. (DOCX) [file pone.0104858.s005.docx]

**Table S5.** Temporal phenotypic stability study of 12 highly resistant DGRP lines.

SD = standard deviation

CV = coefficient of variation

Mean values are percent values based on 2 trials.

| **RAL #** | **Original response** | | | **Response after at least 5 months** | | |
| --- | --- | --- | --- | --- | --- | --- |
|  | *Mean* | *SD* | *CV* | *Mean* | *SD* | *CV* |
| 57 | 86 | 14.14 | 16.44 | 62 | 17 | 27.42 |
| 69 | 95 | 4.24 | 4.46 | 34 | 22.62 | 66.53 |
| 91 | 98 | 2.83 | 2.89 | 80 | 5.6 | 7.00 |
| 142 | 82 | 19.8 | 24.15 | 71 | 12.8 | 18.03 |
| 149 | 50.8 | 21.21 | 41.75 | 22 | 25.4 | 115.45 |
| 208 | 89 | 12.73 | 14.30 | 93 | 1.4 | 1.51 |
| 237 | 57 | 32.53 | 57.07 | 48 | 51 | 106.25 |
| 318 | 49 | 4.24 | 8.65 | 12 | 5.6 | 46.67 |
| 338 | 90 | 14.14 | 15.71 | 27 | 4.2 | 15.56 |
| 378 | 52.5 | 28.28 | 53.87 | 28 | 17 | 60.71 |
| 405 | 52 | 5.66 | 10.88 | 57 | 38.2 | 67.02 |
| 808 | 63 | 9.9 | 15.71 | 58 | 0 | 0.00 |
