# Supplementary material for: Genome-Wide Association Analysis of Radiation Resistance in Drosophila melanogaster
Source: PLoS One. 2014 Aug 14;9(8):e104858. doi: 10.1371/journal.pone.0104858 (PMC4133248; doi:10.1371/journal.pone.0104858)
Supplement: Table S6 — Wolbachia infection status has no signficant effect on the overall DGRP radiation response. (DOCX) [file pone.0104858.s006.docx]

**Table S6.** *Wolbachia* infection (WI) status has no significant effect on the overall DGRP radiation response.

| **Phenotype** | **WI negative lines** | **WI positive lines** | **NA** | **ND** |
| --- | --- | --- | --- | --- |
| Resistant, all | 30 | 32 | 3 | 1 |
| Sensitive | 46 | 46 | 2 | 1 |

WI = *Wolbachia* infection

NA = not available

ND = not determined

Equal number of WI positive and negative sensitive lines, and almost equal numbers in each category for resistant lines, demonstrate that the WI status has no significant effect on radiation response.
