# Supplementary material for: Genome-Wide Association Analysis of Radiation Resistance in Drosophila melanogaster
Source: PLoS One. 2014 Aug 14;9(8):e104858. doi: 10.1371/journal.pone.0104858 (PMC4133248; doi:10.1371/journal.pone.0104858)
Supplement: File S1 — Contains the following files: Figure S1. Variation in radiation response among common Drosophila lab strains and 10 DGRP lines. The lab strains used were Canton S, yw and w1118. The 10 DGRP lines were chosen at random. 50 males from each line were aged for 7–11 days, irradiated at 1382 Gy and the number of survivors were scored 24 hours post-irradiation. The data shown represents the mean of two independent trials. Figure S2. Highly resistant DGRP strains survive over a long period following irradiation. 50 males from RAL-91 and RAL-142 strains were aged for 7–11 days, irradiated with 1382 Gy and scored for survivors over a period of 10 days. (PPT) [file pone.0104858.s011.ppt]

## Slide 1
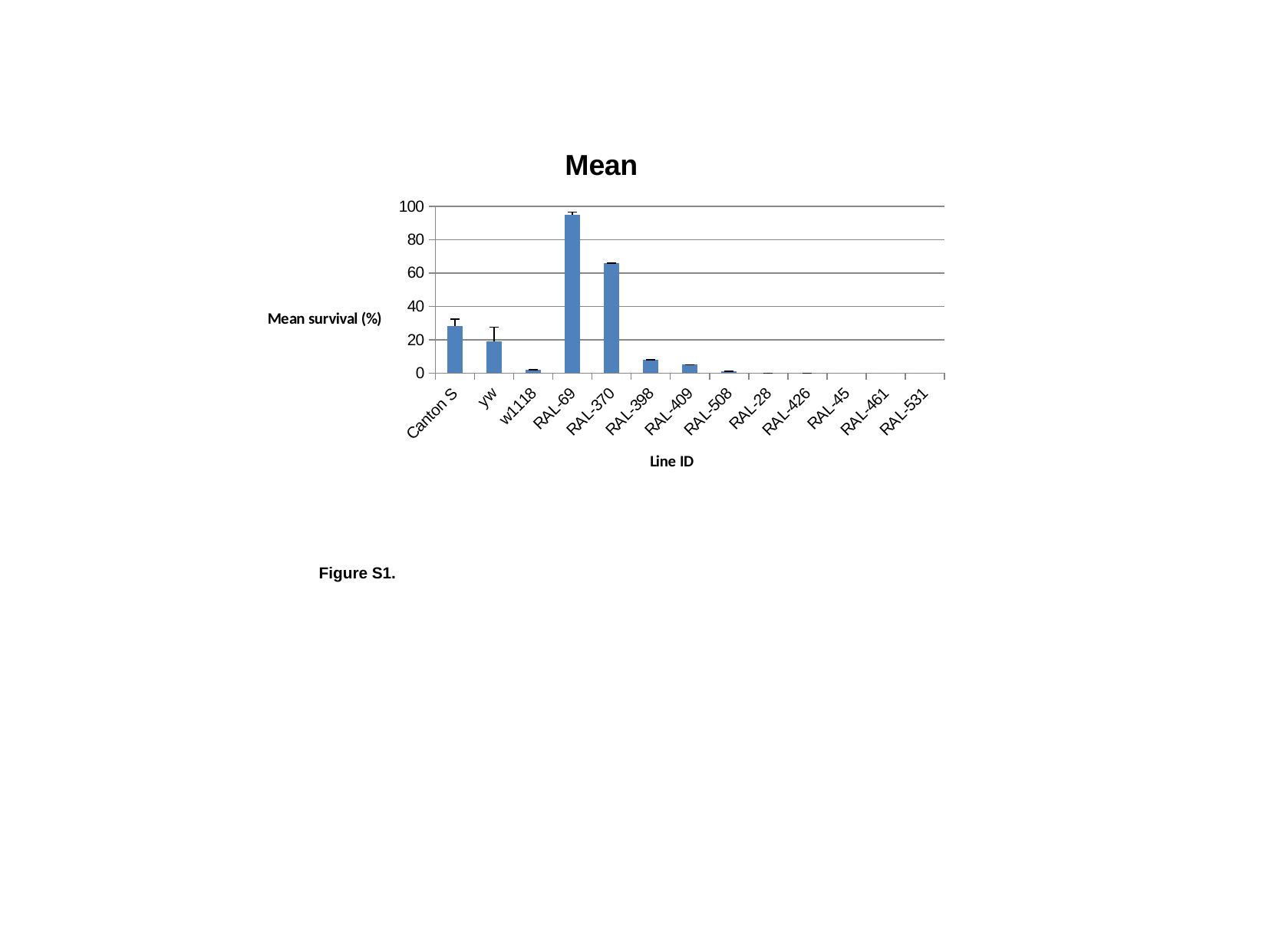

### Chart: Mean
| Category | Mean |
|---|---|
| Canton S | 28.0 |
| yw | 19.0 |
| w1118 | 2.0 |
| RAL-69 | 95.0 |
| RAL-370 | 66.0 |
| RAL-398 | 8.0 |
| RAL-409 | 5.0 |
| RAL-508 | 1.0 |
| RAL-28 | 0.0 |
| RAL-426 | 0.0 |
| RAL-45 | 0.0 |
| RAL-461 | 0.0 |
| RAL-531 | 0.0 |Figure S1.

## Slide 2
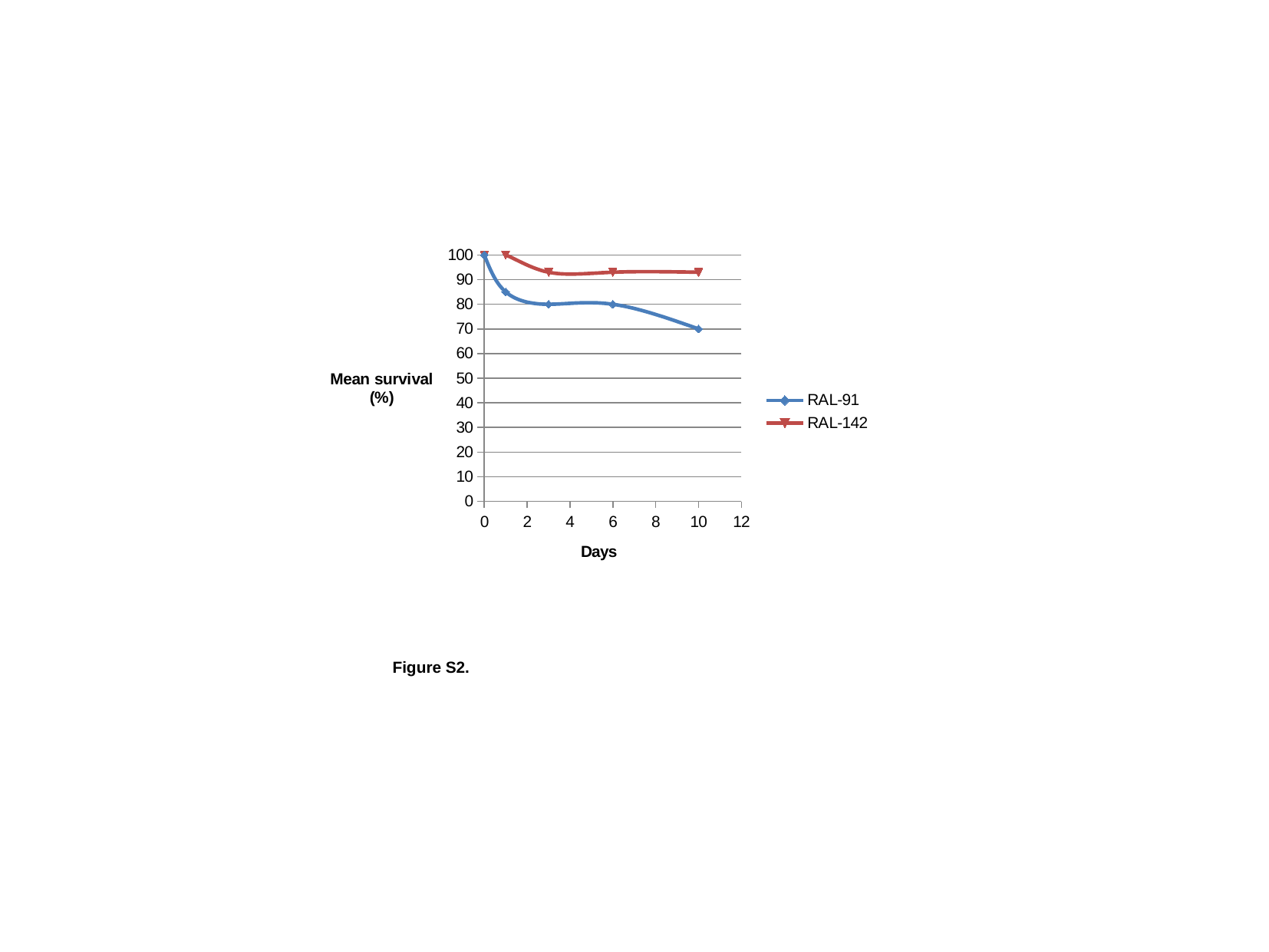

### Chart
| Category | RAL-91 | RAL-142 |
|---|---|---|Figure S2.
